# Golden bile powder prevents drunkenness and alcohol-induced liver injury in mice via the gut microbiota and metabolic modulation

**DOI:** 10.1186/s13020-024-00912-2

**Published:** 2024-03-02

**Authors:** Yarong Wang, Zhenzhuang Zou, Sihua Wang, Airong Ren, Zhaolin Ding, Yingying Li, Yifang Wang, Zhengming Qian, Baolin Bian, Bo Huang, Guiwei Xu, Guozhen Cui

**Affiliations:** 1 School of Bioengineering, Zhuhai Campus of Zunyi Medical University, Zhuhai, 519000 Guangdong China; 2 Department of Pediatrics, The Fifth Affiliated Hospital of Zunyi Medical University, Zhuhai, 519000 Guangdong China; 3 College of Medical Imaging Laboratory and Rehabilitation, Xiangnan University, Chenzhou, 423000 Hunan China; 4 Institute of Chinese Materia Medica, China Academy of Chinese Medical Sciences, Beijing, 100700 China

**Keywords:** Golden bile powder, Drunkenness, Alcoholic liver disease, Network medicine, Metabolomics, Gut microbiota

## Abstract

**Background:**

Drunkenness and alcoholic liver disease (ALD) are critical public health issues associated with significant morbidity and mortality due to chronic overconsumption of alcohol. Traditional remedies, such as bear bile powder, have been historically acclaimed for their hepatoprotective properties. This study assessed the efficacy of a biotransformed bear bile powder known as golden bile powder (GBP) in alleviating alcohol-induced drunkenness and ALD.

**Methods:**

A murine model was engineered to simulate alcohol drunkenness and acute hepatic injury through the administration of a 50% ethanol solution. Intervention with GBP and its effects on alcohol-related symptoms were scrutinized, by employing an integrative approach that encompasses serum metabolomics, network medicine, and gut microbiota profiling to elucidate the protective mechanisms of GBP.

**Results:**

GBP administration significantly delayed the onset of drunkenness and decreased the duration of ethanol-induced inebriation in mice. Enhanced liver cell recovery was indicated by increased hepatic aldehyde dehydrogenase levels and superoxide dismutase activity, along with significant decreases in the serum alanine aminotransferase, aspartate aminotransferase, alkaline phosphatase, triglyceride, and total cholesterol levels (*P* < 0.05). These biochemical alterations suggest diminished hepatic damage and enhanced lipid homeostasis. Microbiota analysis via 16S rDNA sequencing revealed significant changes in gut microbial diversity and composition following alcohol exposure, and these changes were effectively reversed by GBP treatment. Metabolomic analyses demonstrated that GBP normalized the alcohol-induced perturbations in phospholipids, fatty acids, and bile acids. Correlation assessments linked distinct microbial genera to serum bile acid profiles, indicating that the protective efficacy of GBP may be attributable to modulatory effects on metabolism and the gut microbiota composition. Network medicine insights suggest the prominence of two active agents in GBP as critical for addressing drunkenness and ALD.

**Conclusion:**

GBP is a potent intervention for alcohol-induced pathology and offers hepatoprotective benefits, at least in part, through the modulation of the gut microbiota and related metabolic cascades.

**Supplementary Information:**

The online version contains supplementary material available at 10.1186/s13020-024-00912-2.

## Introduction

Alcoholic liver disease (ALD), characterized by the progression from steatohepatitis to fibrosis, cirrhosis, and ultimately hepatocellular carcinoma, is predominantly attributed to chronic, excessive alcohol consumption. The increasing prevalence of ALD mirrors the global increase in alcohol consumption. A significant correlation has been established wherein daily consumption of alcohol above 40 g for more than a year correlated with a 2% increase in the risk of liver cancer, highlighting the role of alcoholic cirrhosis in the onset of hepatocarcinogenesis [1]. The liver, the central organ for alcohol catabolism and metabolization, accounts for approximately 90% of consumed alcohol and is highly vulnerable to alcohol-related harm. Pathological sequelae manifest as various disturbances, including hepatocyte apoptosis, gastric mucosal lesions, compromised immune responses, intensified oxidative stress, and lipid peroxidation [2]. Ethanol, the principal component of alcoholic beverages or liquor, is absorbed and metabolized in the liver, primarily by alcohol dehydrogenase and acetaldehyde dehydrogenase, ultimately being oxidized to water and carbon dioxide. The clinical burden of ALD is significant, highlighting the urgent need for more effective pharmacological and therapeutic strategies. Historically, natural products and their derivatives have made significant contributions to pharmacotherapy [3], suggesting that the development of novel preventative and therapeutic interventions for ALD based on natural compounds is promising.

Natural bear bile is the cornerstone of traditional Chinese medicine and has been used for treating liver dysfunction. Its primary active components include bile acids, cholesterol, bilirubin, amino acids, proteins, and trace metallic elements. Contemporary studies have revealed that bear bile powder exhibits various pharmacological effects with minimal toxicological effects, and its purified compounds have been used for treating hepatic and biliary conditions for several decades [4]. Nonetheless, ethical and conservation concerns significantly complicate the procurement of natural bear bile. To address this issue, a novel methodology was developed to replicate bear bile acid enterohepatic circulation in vitro, by employing chicken bile and a dual-enzyme transformation process, ultimately yielding a synthetic bear bile powder [5], referred to as golden bile powder (GBP).

Comparative analyses revealed that the chemical and biological profiles of GBP exhibited remarkable congruence with those of high-grade natural bear bile powder, exhibiting compositional fidelity ranging from 96.1% to 97.6%, with an overall content similarity surpassing 95.5%. Prompted by these findings, we employed an ALD mouse model to investigate the therapeutic potential of GBP. The objective of this research was to investigate the ability of GBP to ameliorate the effects of drunkenness and to offer protection against alcohol-induced hepatic injury, thereby laying a robust scientific foundation for the advancement of GBP-derived therapeutic applications.

## Materials and methods

### Chemicals and reagents

The composition of Macau Xiong Yantang gold bile powder capsule, named GBP, was obtained from China Goldenbile Biological Technology Co., Ltd. The positive control drug metadoxine was supplied by Shandong Qidu Pharmaceutical Co., Ltd. Biochemical assay reagents, including alanine aminotransferase (ALT), aspartate aminotransferase (AST), alkaline phosphatase (AKP), total cholesterol (TC), triglycerides (TG), superoxide dismutase (SOD), and acetaldehyde dehydrogenase (ALDH) detection kits, were all purchased from Nanjing Jiancheng Bioengineering Institute (Nanjing Jiancheng Bioengineering Institute, Nanjing, China).

### Animals and treatment

Male C57BL/6J mice aged 6–8 weeks were obtained from Guangdong Medical Laboratory Animal Centre and maintained under previously described conditions [6]. All methods described in this study were reviewed and approved by the Ethics Committee of the Zhuhai Campus of Zunyi Medical University. The mice were randomly divided into five groups: the control group, vehicle group, positive control group (300 mg/kg metadoxine), low-dose GBP group (100 mg/kg), and high-dose GBP group (400 mg/kg). Each group consisted of 10–13 animals. The control and vehicle groups were administered water for the first 14 days, while the intervention groups were administered the corresponding test substances by gavage. On the 15th day, 30 min post-administration, 50% ethanol was given via gavage (0.1 mL per mouse). The latency period of drunkenness in mice was recorded from the time of alcohol administration to the onset of the righting reflex, and the duration of drunkenness was measured from the onset of the righting reflex to its disappearance. Following the final alcohol administration, food was withheld, but water was provided for 12 h, after which dissection and tissue collection were performed. Subsequently, blood samples, liver tissues, and fecal samples were collected for further analysis.

### Observation of liver tissue histopathological sections

The major lobe of the liver was fixed in 4% formaldehyde for 24 h. The liver tissues were then longitudinally sectioned into approximately 4 mm slices, dehydrated, embedded, and stained with hematoxylin and eosin (H&E). Histopathological alterations were observed under a microscope (Olympus BX50, Tokyo, Japan) and evaluated using an acute pathological scoring system as described previously [7]. Quantitative analysis was performed using ImageJ software (version 1.8.0, NIH, Bethesda, MD).

### Detection of biochemical markers in mouse serum and liver

Mouse serum samples were thawed on ice and analyzed according to the manual of various kits for determining the serum levels of ALT, AST, AKP, TC, and TG. Liver tissues weighing 0.1 g were homogenized in 0.9 mL of PBS and centrifuged at 10,000 ×*g* at 4 °C for 10 min. The supernatant was collected, and the protein concentration and SOD content in the supernatant were determined according to the instructions of the kit. ALDH content in liver tissue was evaluated via an ELISA kit.

### Serum sample preparation for mass spectrometry analysis

Serum samples were gently thawed on ice to preserve integrity. For the preparation of each sample, 100 μL of serum was meticulously combined with 300 μL of a chilled methanol: acetonitrile solution (2:1, v/v). The mixture was vortexed for 60 s to induce protein precipitation, followed by centrifugation at 3000 ×*g* and 4 °C for 10 min to clarify the solution. The supernatant was then passed through a 0.22 μm filter to remove any particulate matter before being subjected to ultra-high performance liquid chromatography coupled with quadrupole time-of-flight mass spectrometry (UHPLC-Q-TOF/MS, Waters Corp., Milford, USA) for analysis.

### Mass spectrometry conditions

Chromatographic separation of the samples was performed using a Waters Acquity™ ultra-performance LC system (Waters Corp., Milford, USA). The instrumentation and conditions were set up as previously described [8]. Briefly, chromatographic separation was achieved using an ACQUITY UPLC BEH C18 column (2.1 mm × 50 mm, 1.8 μm) maintained at 40 °C. The mobile phase comprised a 0.1% formic acid aqueous solution (solvent A) and acetonitrile (solvent B). A flow rate of 0.3 mL/min was applied with the following linear gradient program: 0–1 min, 5 → 20% B; 1–5.5 min, 20% → 25% B; 5.5–6 min, 25% → 30% B; 6–8 min, 30% → 30% B; 8–9 min, 30% → 35% B; 9–17 min, 35% → 65% B; 17–18 min, 65% → 100% B; 18–19 min, 100% B; 19–19.1 min, 100% → 5% B; 19.1–20 min, 5% B. The injection volume for each sample was 2 μL.

A Waters SYNAPT XS mass spectrometer (Waters Corp., Milford, USA) was connected to the UPLC system via an ESI source, which was operated in both positive and negative ionization modes. Spectra were recorded in MSE acquisition mode, covering a mass range from 50 to 1200 Da. For MS/MS analysis, precursor ions were fragmented using collision energies ranging from 20 to 50 eV, and each spectrum had a scan time of 0.5 s. The cone voltage was set at 40 kV, and the capillary voltage was set at 2.0 kV. The desolvation gas was flowed at 600 L/h at a high temperature of 350 °C, and the source temperature was maintained at 120 °C with a cone gas flow of 50 L/h. Precise mass measurements were ensured through lock mass calibration using [M + H]^+^ (m/z 556.2771) in positive mode and [M-H]^−^ (m/z 554.2615) in negative mode, derived from leucine enkephalin. The data acquisition and analysis were conducted using MassLynx™ V4.1 software (Waters Corp., Milford, MA, USA).

Metabolomic analysis and data preprocessing were conducted using Progenesis QI V 2.1 software (Waters Corp., Milford, MA, USA). Principal component analysis (PCA) and orthogonal partial least squares discriminant analysis (OPLS-DA) were performed with EZinfo V 3.0 software (MKS Umetrics, Umea, Sweden). Additionally, the variable importance in projection (VIP) value was obtained from OPLS-DA. Potential metabolites were selected based on VIP > 1, FC > 2 and *P* < 0.05. The identification of potential metabolites typically involves searching for specific features, often represented by their accurate masses, in databases such as KEGG (https://www.kegg.jp/) and HMDB (http://www.hmdb.ca/). Pathway enrichment and metabolic pathway analysis were carried out using MetaboAnalyst 5.0 (http://metpa.metabolomics.ca). Heatmap visualization and Spearman correlation analysis were conducted using OmicShare bioinformatics platform (http://www.omicshare.com/tools).

### Detection of the gut microbiota

The gut microbiota was profiled through the dissection and mincing of cecal tissues from mice as described previously [9]. Genomic DNA was extracted from each fecal sample using a DNA Extraction Kit, and the purity and concentration of DNA were determined using a NanoDrop One spectrophotometer (Thermo Fisher Scientific, Waltham, MA, USA). The V3-V4 region of the 16S rRNA gene was PCR amplified with barcode-specific primers and TaKaRa Premix Taq^®^ version 2.0 (TaKaRa Biotechnology Co., Dalian, China). Following amplification and verification, the concentrations of the PCR products were determined using GeneTools Analysis Software (version 4.03.05.0; SynGene, Cambridge, UK), and the volume of each sample was calculated based on an equimolar pooling principle. Library construction was then performed according to the standard protocol of the NEBNext^®^ Ultra^™^ II DNA Library Prep Kit for Illumina^®^ (New England Biolabs, USA). Amplicon libraries were sequenced on the Illumina NovaSeq 6000 platform using the PE250 sequencing method (Guangdong Magigene Biotechnology Co., Ltd., Guangzhou, China). Sequences with ≥ 97% similarity were clustered into operational taxonomic units (OTUs) using USEARCH (http://www.drive5.com/usearch/), and taxonomic annotations were assigned using the SILVA database (https://www.arb-silva.de/), with a confidence threshold score of ≥ 0.5.

### Microbial bioinformatics analysis

Venn diagrams were generated to depict core and shared gut microbiota species across different mouse groups. Bacterial taxonomic classifications and clustering heatmaps were generated to represent the bacterial abundance within various groups. The beta diversity of the mouse gut microbiota was analyzed via principal coordinates analysis (PCoA). In addition, to identify distinct taxa, we applied the linear discriminant analysis effect size (LEfSe) method for biomarker discovery. Non-parametric Kruskal–Wallis (KW) rank tests and pairwise Wilcoxon tests were utilized to detect species with significant differences between groups. Subsequently, linear discriminant analysis (LDA) was conducted to assess the effect size of each differentially abundant taxonomic unit. Finally, species with LDA scores (log10) ≥ 3.5 were defined as biomarkers.

### Collection of GBP ingredients and metabolite targets

To identify the targets of the ingredients and various metabolites of GBP, we input the names of each chemical ingredient and metabolite detected by MS spectra into seven distinct databases to search for “drug targets”. The interaction data between the ingredients and targets were sourced from BindingDB [10], DrugBank [11], Ingenuity Pathway Analysis (IPA) [12], Therapeutic Target Database (TTD) [13], IUPHAR Guide to IMMUNOPHARMACOLOGY [14], Comparative Toxicogenomics Database (CTD) [15] and HIT2.0 [16]. Our network analysis focused solely on protein targets with experimental evidence documented in the literature.

### Manual curation of human drunkenness and ALD-associated genes

This study collected genes associated with drunkenness and ALD as defined by Medical Subject Headings (MeSH) and the Unified Medical Language System (UMLS). We integrated disease-related gene annotation data from various databases: Comparative Toxicogenomics Database (CTD) [15], GeneCards [17], DisGeNET [18], and Online Mendelian Inheritance in Man (OMIM) [19]. To ensure research quality, the collected genes were filtered, and only those genes with a proven direct relationship in the experiments were retained. The Entrez gene ID for each gene was obtained from NCBI and stored in CSV format for subsequent data processing as described previously [8].

### Calculation of network proximity

We constructed a human protein–protein interaction network based on 18 databases and the literature [20–23]. It comprises 18,375 proteins (nodes) and 485,412 edges. Following a previously described method [8, 24], we calculated the network proximity between drunkenness and GBP ingredients, as well as between ALD and GBP ingredients. The network proximity was measured using the closest distance, reflecting the path length between disease-related genes and the protein targets of the ingredients. In the formula, S represents genes related to drunkenness or ALD, T represents protein targets of ingredients, and ${d}_{c}(S,T)$ signifies the closest distance between S and T within the human protein–protein interaction network. The network proximity of S and T was calculated using the following equation.

$${d}_{c}(S,T)=\frac{1}{\parallel T\parallel }\sum_{t\in T} \underset{s\in S}{min} d(s,t)$$

To assess the statistical significance of the network distance between drug components and diseases, we constructed a reference distance distribution as described previously [24]. This distribution was derived by calculating the network proximity between 1000 pairs of randomly selected protein groups with size and degree distributions similar to those of the original disease-related genes and drug targets. We then calculated the average distance ${\mu }_{{d}_{c}}(S,T)$ and standard deviation ${\sigma }_{{d}_{c}}(S,T)$ by using these 1000 expected distances. The Z value is calculated by Equation. A Z value less than zero with *P* < 0.05 was considered to indicate statistical significance. Notably, the smaller the Z value is, the closer the distance between the drug and the disease. To enhance the accessibility of computational analyses in network proximity, we have developed an online computational platform, which can be accessed at www.zmupredict.cn. This platform enables users to freely submit data for an array of calculations associated with network medicine. The corresponding targets (proteins) of the ingredients and disease in the PPI were extracted and subsequently imported into Gephi 0.9.2 software (https://gephi.org) for network visualization analysis as described previously [25].

$${Z}_{{d}_{c}}=\frac{d(S,T)-{\mu }_{{d}_{c}}(S,T)}{{\sigma }_{{d}_{c}}(S,T)}$$

### Statistical analysis

The data were statistically analyzed using GraphPad Prism 9.0. The results are expressed as the mean ± standard deviation (SD). One-way ANOVA followed by Tukey’s multiple comparisons test was used. Differences were considered statistically significant at *P* < 0.05.

## Results

### The effects of GBP on the latency and duration of drunkenness in mice

**Fig. 1:**
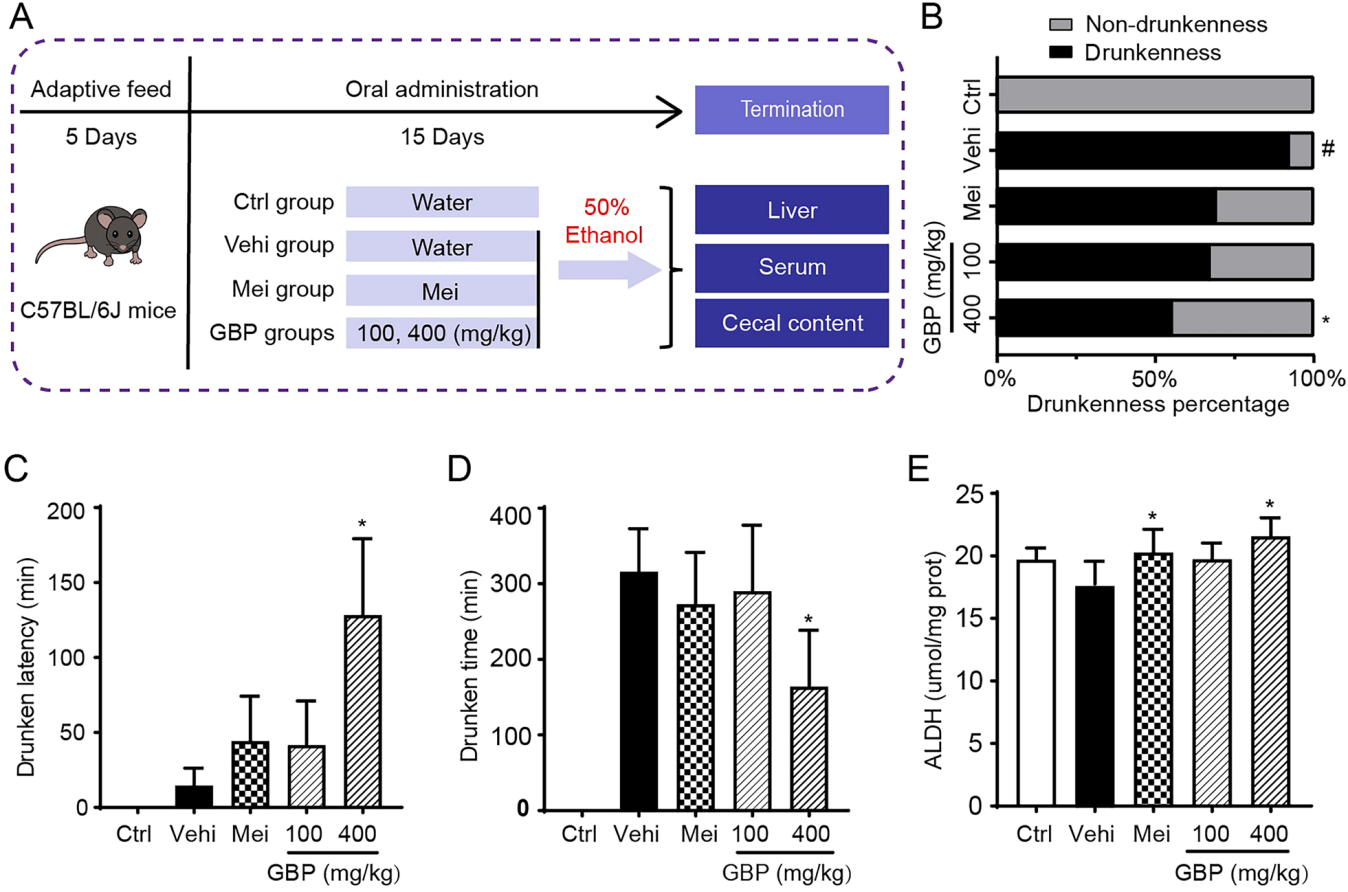
GBP prevents drunkenness in mice. **A** Study design for assessing the impact of GBP in alcohol treated mice. **B** Reduced incidence of drunkenness in GBP treatment groups. **C** Increased latency to drunkenness with GBP administration in mice. **D** Duration of drunkenness decreased upon GBP treatment. **E** Increased ALDH enzyme levels following GBP exposure. The data are expressed as the mean ± SD (n = 10–13). Compared with the control group, ^#^*P* < 0.05; compared with the vehicle, *P < 0.05

The methodology of GBP for the prevention of drunkenness and ALD is depicted in Fig. 1A, which outlines the experimental protocol. As shown in Fig. 1B, 92% of the vehicle group exhibited drunkenness, whereas treatment with metadoxine (300 mg/kg) resulted in a 69% reduction in the incidence of drunkenness. Similarly, compared with those in the vehicle group, the proportions of mice in the low-dose (100 mg/kg) and high-dose (400 mg/kg) GBP groups decreased to 67% and 55%, respectively, indicating dose-dependent decreases in drunkenness rates of 22%, 25%, and 37%, respectively, compared to those in the vehicle group. As shown in Fig. 1C, the latency to drunkenness was 14.53 ± 11.73 min in the vehicle group and 44.25 ± 30.03 min in the positive control group. The low- and high-dose GBP groups demonstrated an even more pronounced extension of latency periods, 41.63 ± 29.43 and 128.2 ± 65.08 min, respectively. This represents latency periods increased by a factor of approximately 2.86 and 8.83 compared to those of the vehicle group. Furthermore, the duration of drunkenness, which was measured from the loss of the righting reflex, was noted at 315.73 ± 57.00 min in the vehicle group. In contrast, the positive control group experienced a significant reduction in duration to 272.93 ± 68.51 min, constituting a 14% decrease. The low- and high-dose GBP groups had durations of 290.03 ± 87.37 min and 163.98 ± 74.53 min, respectively, marking reductions of 8% and 48%, respectively (Fig. 1D). Similarly, compared with those in the vehicle group, the hepatic ALDH enzyme levels in the positive control group were by 14%, while those in the low- and high-dose GBP groups were by 11% and 21%, respectively (Fig. 1E). GBP treatment at high doses (400 mg/kg) led to noteworthy outcomes, as depicted in Fig. 1B, C, D, E. There was a significant decrease in the percentage of drunkenness, an extension in the latency period for drunkenness, a reduction in the duration of drunkenness, and an increase in hepatic ALDH enzyme levels in GBP treatment (400 mg/kg) group compared to the vehicle group (*P* < 0.05). Collectively, these findings affirm the potency of GBP in curtailing alcohol-induced impairment, underscoring its preventive efficacy against drunkenness and ALD.

### Effects of GBP on ALD in mice

**Fig. 2:**
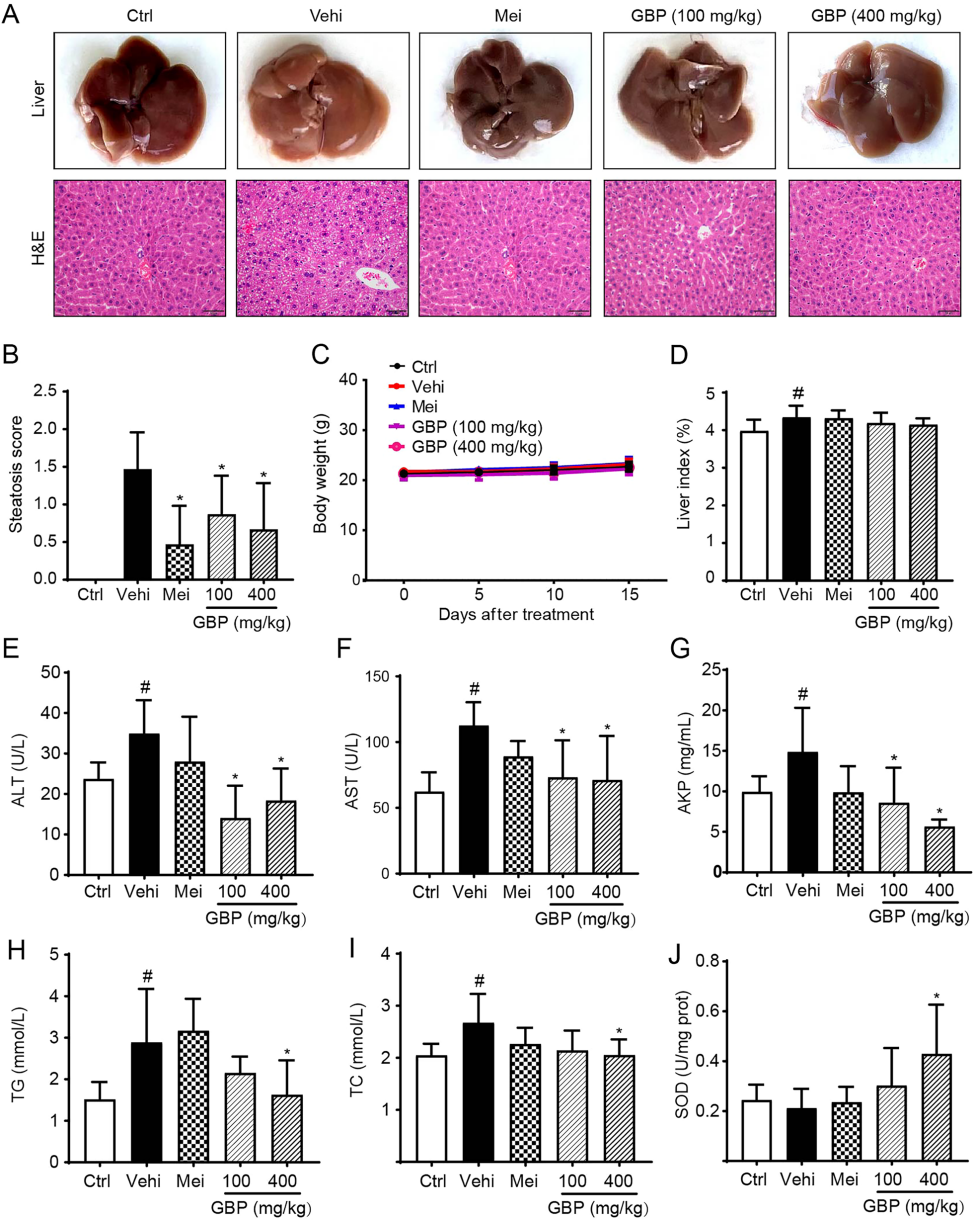
GBP ameliorates alcohol-induced liver injury in mice. **A** Representative liver tissue morphology and histopathological sections. **B** Quantification of liver tissue pathological score, **C** body weight, and **D** liver index. The serum levels of ALT **E**, AST **F**, AKP **G**, TG **H**, TC **I**, and the activity of SOD in liver tissues **J** were evaluated. The data were expressed as the mean ± SD (n = 10–13). Compared with the control group, ^#^*P* < 0.05; compared with the vehicle, *P < 0.05

Pathological changes in the liver are one of the primary organs reflecting the safety of a drug. To determine the protective effect of GBP on acute alcoholic liver injury, the following pathological analysis was conducted. As shown in Fig. 2A, the livers of mice in the blank group appeared healthy and normal with neatly arranged hepatocytes, no vacuolar lesions, no inflammatory cell infiltration, and no necrosis. In contrast, compared with those in the control group, the vehicle group exhibited disorganized hepatocyte arrangement, significant cell swelling, blurred cell boundaries, and an increased number of necrotic hepatocytes and fatty vacuoles. The liver scoring results are shown in Fig. 2B. Compared with those in the vehicle group, the positive control group and GBP group exhibited significant improvements in terms of well-preserved liver cell structure, clear cell boundaries, and almost no vacuolar or necrotic changes (*P* < 0.05). The mouse body weight data, as shown in Fig. 2C, indicated that the body weight steadily increased during the experiment. When acute liver injury occurs, the liver exhibits noticeable swelling. As shown in Fig. 2D, compared with that in the control group, the liver index of the vehicle group was significantly increased (*P* < 0.05), and GBP slightly reversed these changes but not significantly (*P* > 0.05) compared to those in the control group. Collectively, our results demonstrated that GBP effectively improved alcoholic liver injury in mice.

### Effects of GBP on the serum and liver biochemical marker levels in ALD mice

To ascertain the effect of GBP on serum biochemical markers in a mouse model of ALD, we evaluated biochemical indicators such as AST, ALT, AKP, TG, TC, and hepatic SOD. The serum ALT, AST and AKP levels, which are principal biochemical markers reflecting liver function, were significantly greater in the vehicle group than in the control group (*P* < 0.05), confirming the successful establishment of the ALD model in mice. In contrast, GBP group exhibited significant reductions in ALT, AST and AKP levels (Fig. 2E, F and G, P < 0.05). TG and TC, key indicators of lipid changes in the liver, were significantly elevated in the vehicle group compared to those in the control group (*P* < 0.05), indicating that alcohol disrupts lipid metabolism, leading to lipid accumulation. A high dose of GBP significantly decreased the levels of TG and TC (Fig. 2H, I, *P* < 0.05), suggesting that GBP may accelerate triglyceride metabolism to reduce fat accumulation. SOD, an antioxidative biomarker, reflects the capacity of tissue to resist oxidative damage. As illustrated in Fig. 2J, the antioxidative capacity was significantly enhanced in the high-dose GBP group than in the vehicle group (*P* < 0.05). Our findings indicate that GBP possesses a robust hepatoprotective effect on ALD mice.

### The gut microbiota landscape and its correlation with liver biochemistry

**Fig. 3:**
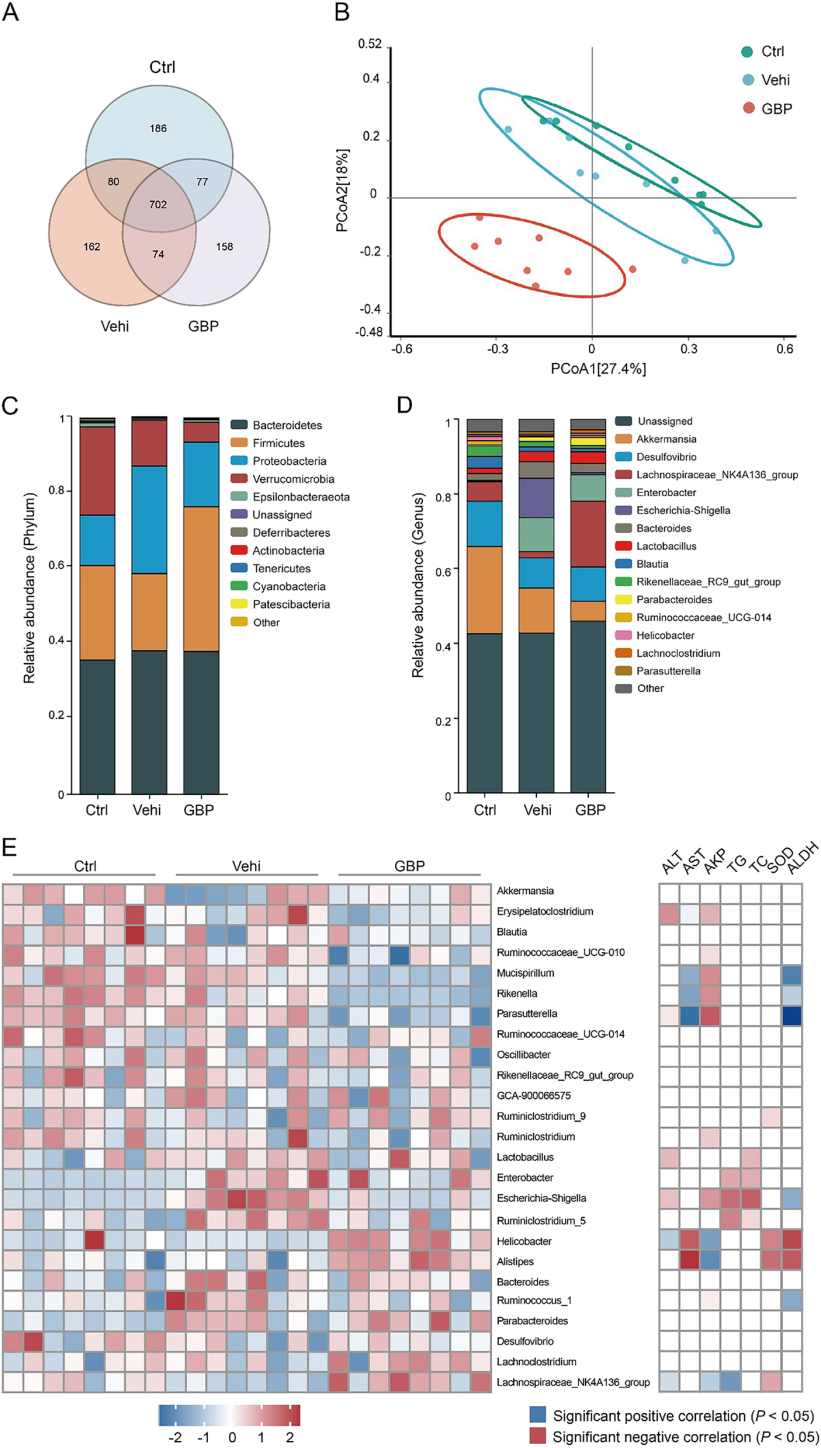
Modulation of the gut microbiota and correlation with liver biochemistry in response to GBP treatment. **A** Venn diagram showing shared and unique OTUs among the control, Vehi, and GBP groups. **B** PCoA plot visualizing beta diversity, with greater distances indicating more distinct microbiota compositions. **C** Bar chart detailing the relative abundance of microbial taxa at the phylum level among the different groups. **D** Bar chart displaying the relative abundance at the genus level. **E** Heatmap of genus-level abundance and correlation with key hepatic biochemical parameters. The sample size of each group was 8. The blue squares indicate a significant positive correlation, and the red squares indicate a significant negative correlation (*P* < 0.05)

To examine changes in the gut microbiome composition in mice after GBP treatment, we sequenced the V3-V4 region of the 16S rRNA gene from fecal samples. Venn diagrams were constructed to analyze the commonalities in microbial richness across the groups. As depicted in Fig. 3A, there was an overlap of 702 OTUs among the three groups: 1045 OTUs in the control group, 1018 OTUs in the vehicle group, and 1011 OTUs in the GBP group. Beta diversity was analyzed using PCoA. As shown in Fig. 3B, the gut microbial communities of the mice that consumed ethanol were different from those of the control group or the vehicle group. Concurrently, PCoA revealed a distinct separation pattern of gut microbial communities in mice treated with GBP. At the phylum level, Bacteroidetes, Firmicutes, Proteobacteria, and Verrucomicrobia were the predominant phyla (Fig. 3C). Compared to those in the control group, the vehicle group exhibited increased relative abundances of Proteobacteria and Patescibacteria, whereas the relative abundances of Firmicutes, Epsilonbacteraeota, Actinobacteria, Tenericutes, and Cyanobacteria were lower. However, GBP reversed this trend. At the genus level, the relative abundances of Bacteroides, Enterobacter, and Escherichia-Shigella in the vehicle group exhibited an increase compared to those in the control group, while the relative abundances of Desulfovibrio and Lachnospiraceae NK4A136_group showed a reduction. However, the administration of GBP reversed these changes (Fig. 3D). We identified 25 taxonomic groups at the gene level within the gut microbiota that were significantly influenced by GBP. Among these 25 microbial taxa, Escherichia-Shigella was found to be significantly enriched in the vehicle group and was positively correlated with ALT, AKP, TG and TC but negatively correlated with ALDH. On the other hand, the abundance of Lachnospiraceae NK4A136 decreased significantly in the vehicle group, with negative correlations with ALT, AKP and TG and a positive correlation with SOD. However, supplementation with GBP reversed these changes (Fig. 3E). These data collectively suggest that GBP modulates the gut microbiota, which may be correlated with improvements in hepatic function.

**Fig. 4:**
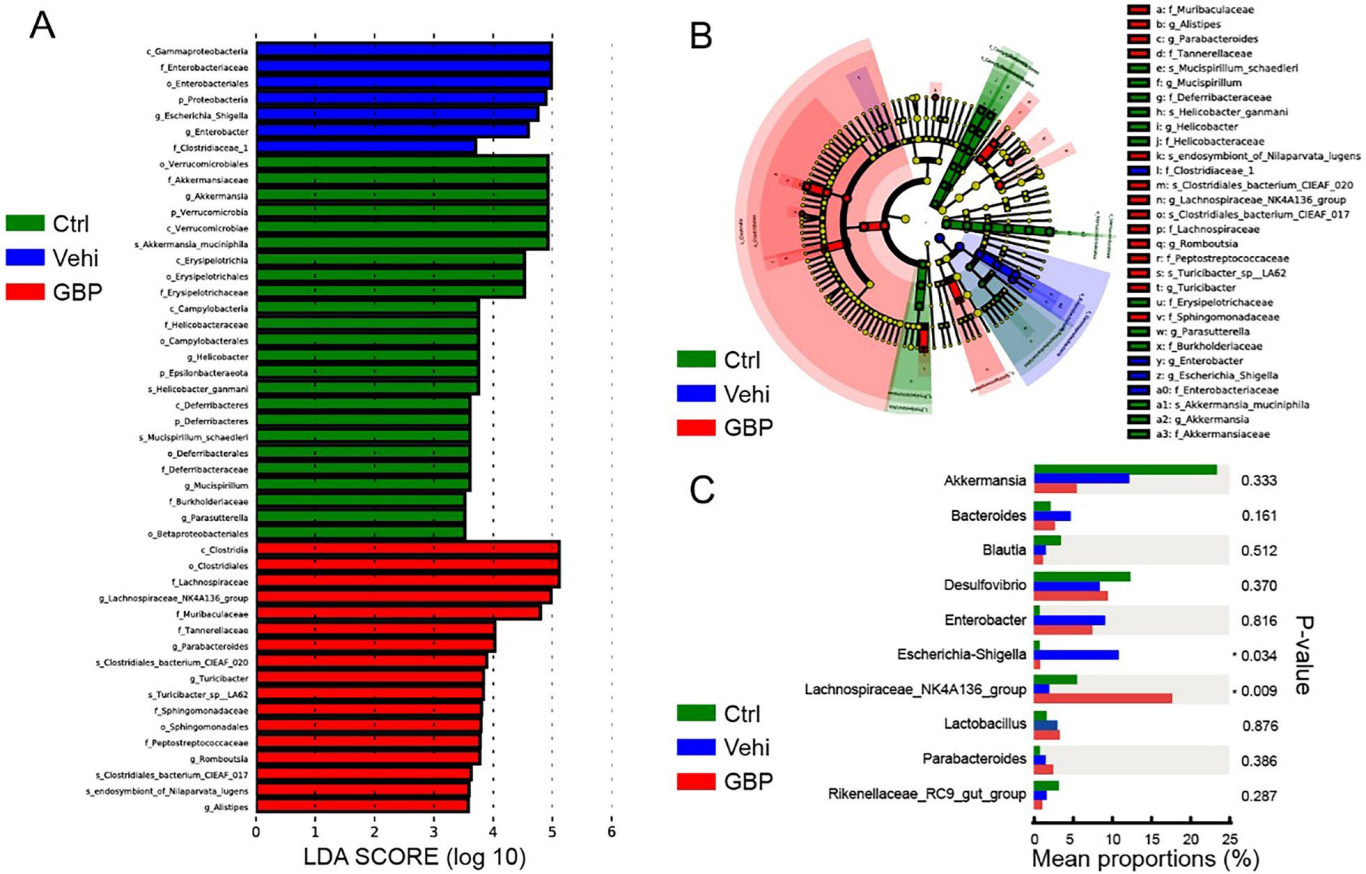
Comparative analysis of microbial communities among the groups. **A** The histogram displays LDA scores for features with differential abundance among the various groups. The scores indicate the magnitude of the effect size corresponding to each feature in distinguishing among groups. Taxa listed on the y-axis with LDA scores exceeding 3.5 are highlighted as potential biomarkers for their respective groups. **B** A cladogram illustrating the phylogenetic distribution of the microbiota determined by LEfSe analysis, with circle layers representing taxonomic classifications from kingdom to genus. Nodes are color-coded based on the group with the most pronounced association with each taxon. **C** A bar graph shows the statistical evaluation of genus-level differences among the control, vehicle, and GBP groups. The sample size in both groups was 8. Compared with the control group, ^#^*P* < 0.05; compared with the vehicle, *P < 0.05

To elucidate the key active microbial taxa in our study, we employed LEfSe analysis to identify biomarkers at various taxonomic levels (with LDA scores [− log10] > 3.5). Figure 4A reveals the identification of 24, 7, and 17 distinct biomarkers in the control, vehicle, and GBP groups, respectively. Figure 4B illustrates the distribution of different species across levels, with the size of each circle indicating species richness. At the phylum level, biomarkers identified in the control group included Verrucomicrobia, Epsilonbacteraeota, and Deferribacteres, whereas Proteobacteria emerged as a significant biomarker in the vehicle group. Notably, the GBP group exhibited no discernible phylum-level differences. At the genus level, the control group was characterized by the presence of biomarkers such as Akkermansia, Helicobacter, Mucispirillum, Parasutterella, and Enterorhabdus. In contrast, the biomarkers identified in the vehicle group were limited to Escherichia_Shigella and Enterobacter, while the biomarkers identified in GBP group included Lachnospiraceae NK4A136_group, Parabacteroides, Turicibacter, Romboutsia, and Alistipes. Statistical analysis of the intergroup differences revealed that alcohol consumption significantly decrased the community richness of the Lachnospiraceae NK4A136 group and increased the abundance of Escherichia-Shigella. In contrast, GBP administration markedly promoted intestinal microbial homeostasis, as demonstrated in Fig. 4C.

### Identifying the ingredients of GBP in mouse serum via mass spectrometry

**Fig. 5:**
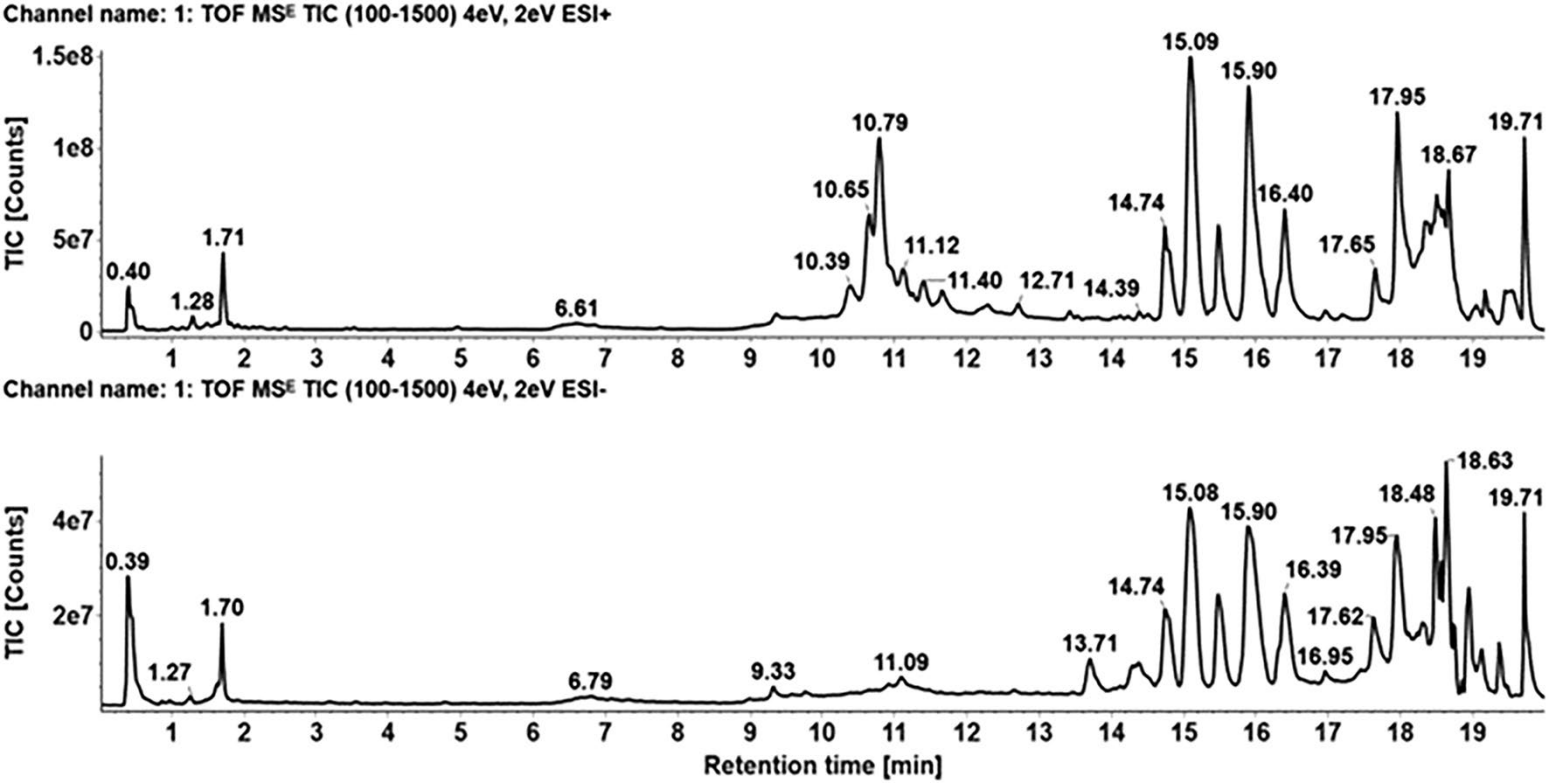
UPLC-Q/TOF–MS chromatographic analysis of GBP ingredients and metabolites. The chromatograms display the retention times (min) of each peak on the x-axis, with the y-axis indicating ion counts that signify the relative abundance of each ingredient or metabolite. The upper chromatogram shows data in positive ion mode, whereas the lower chromatogram shows results in negative ion mode

The UNIFI 1.8.2 (Waters Corp., Milford, USA) platform was utilized to process and analyze the MS data, involving database matching, mass accuracy, isotope patterns, and fragmentation pathways (sourced from the literature and public data). The total ion chromatogram of GBP is shown in Fig. 5. In total, 7 ingredients (3-keto tauroursodeoxycholic acid, 7-ketodeoxycholic acid, cholic acid, taurocholic acid, taurochenodeoxycholic acid, ursodeoxycholic acid, and chenodeoxycholic acid) were identified, and their mass spectrometry details are provided in Additional file 1: Table S1.

### Multivariate statistical analysis and metabolite identification

**Fig. 6:**
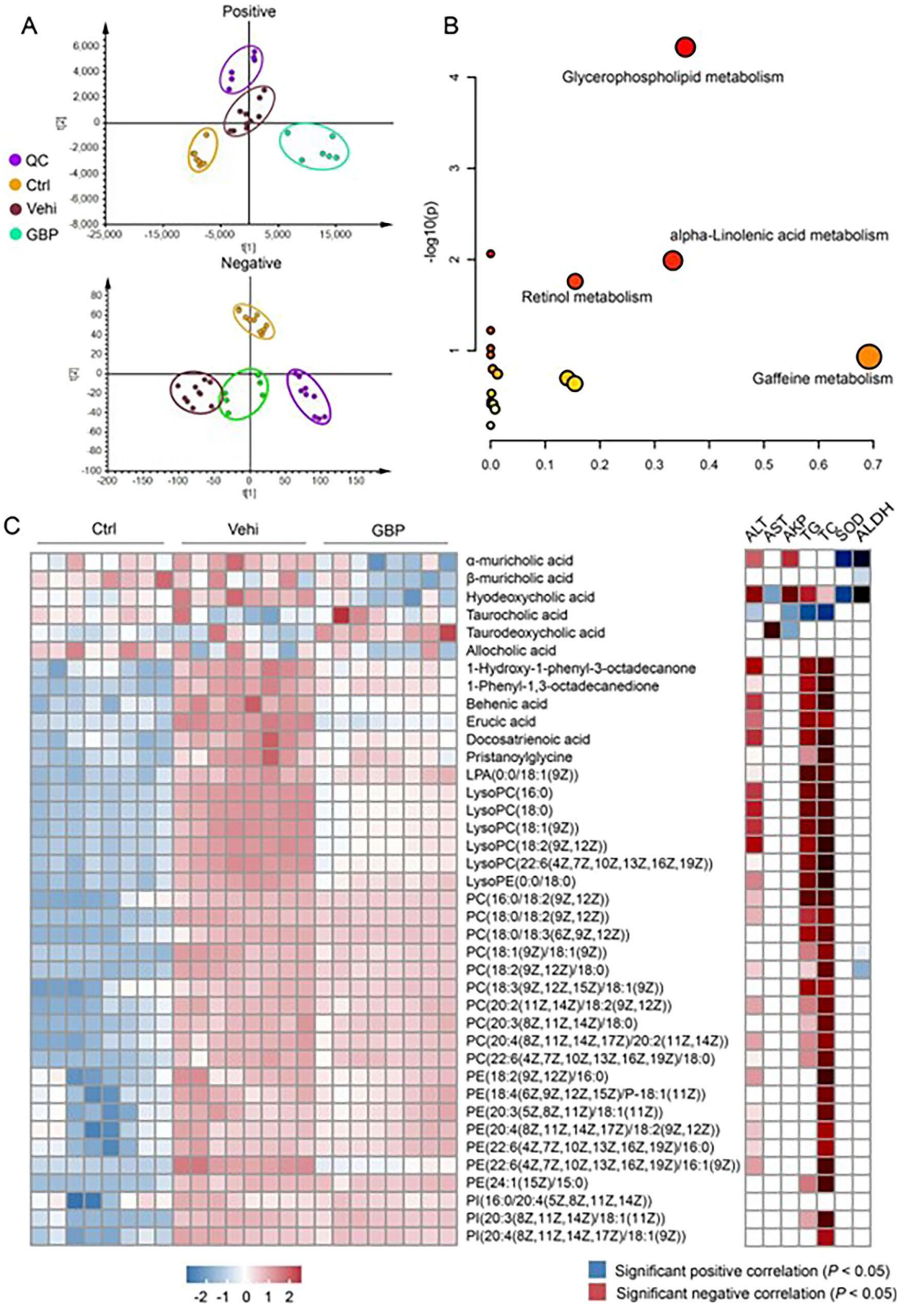
Metabolic profiling and correlation with hepatic biochemical indices in response to treatment. **A** OPLS-DA score plots illustrating the metabolic differences between the control (green), vehicle (blue), and GBP (orange) groups in negative and positive ionization modes. **B** A volcano plot showing metabolites exhibiting significant intergroup differences, with the size reflecting the magnitude of change and the color denoting *P*-value indicating statistical significance. **C** A heatmap representing z-score normalized metabolite intensities in the different groups and their correlation with hepatic biochemistry parameters (ALT, AST, AKP, TG, and TC). The sample size of each group was 8. The blue squares indicate a significant positive correlation, and the red squares indicate a significant negative correlation (*P* < 0.05)

The OPLS-DA score plots distinctly segregated the control, vehicle, and GBP treated groups (Fig. 6A). We identified 39 potential metabolic features significantly influenced by GBP (*P* < 0.05, FC > 2, and VIP > 1). Four crucial metabolic pathways, namely glycerophospholipid metabolism, alpha-linolenic acid metabolism, retinol metabolism, and caffeine metabolism, were identified based on − log (P) > 1 and an impact value > 0.02 (Fig. 6B). Among these 39 metabolites (Additional file 2: Table S2), phospholipids (PCs, PEs, PIs, LysoPCs, and LysoPEs) were significantly enhanced in the vehicle group and positively correlated with TC and TG. Moreover, fatty acids (behenic acid, erucic acid, and docosatrienoic acid) were significantly decreased in response to GBP treatment and were positively correlated with ALT, TG, and TC. Additionally, two bile acids, alpha-muricholic acid (α-MCA) and hyodeoxycholic acid (HDCA), were positively correlated with the levels of ALT and AKP. In contrast, the concentrations of SOD and ALDH were negatively associated with these compounds. These parameters increased in the Vehicle group, but GBP treatment significantly reversed this trend. Furthermore, Taurocholic acid (TCA) was negatively correlated with the contents of ALT, AKP, TG, TC, which decreased in the vehicle mice, respectively (Fig. 6C). Taken together, these results suggest that GBP intervention alters the serum metabolite levels and is correlated with the regulation of biomarkers of hepatic function.

### Correlations between various metabolites and the gut microbiota

**Fig. 7:**
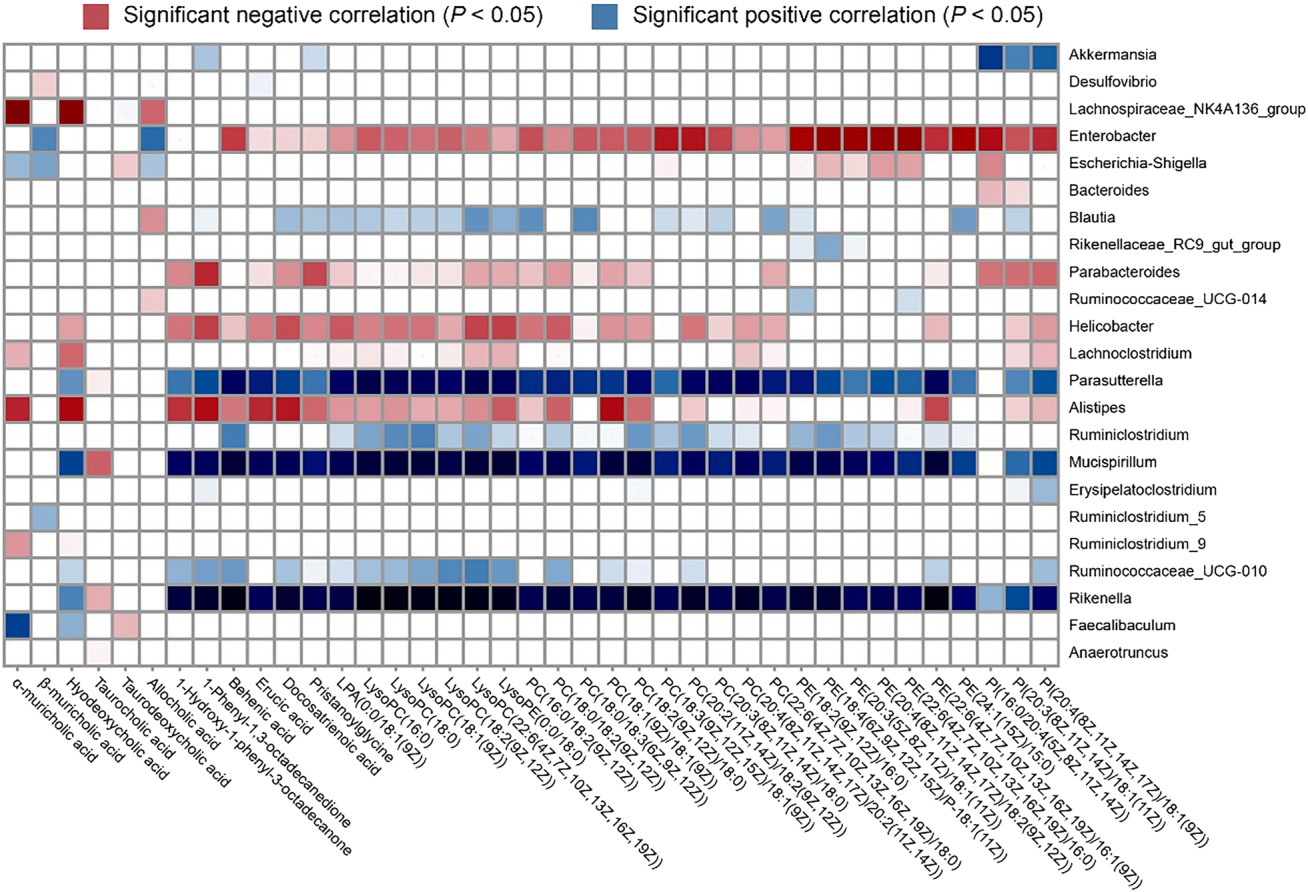
Heatmap visualizing the correlations between metabolites and the gut microbiota. The heatmap employs Spearman’s rank correlation coefficients to display the relationships, with color gradations indicating the correlation strength: red signifies a positive correlation, blue signifies a negative correlation, and the color saturation denotes the magnitude, with deeper hues indicating stronger correlations (*P* < 0.05). The sample size of each group was 8

The potential correlation between the gut microbiota and metabolites was investigated in depth by calculating Spearman’s rank correlation coefficient. A correlation analysis between the 39 differentially abundant metabolites and the 29 distinct microbiota communities at the genus level was performed. This analysis revealed significant correlations (*P* < 0.05) between bacteria and different metabolites, which were visually represented in a heatmap (Fig. 7). Rikenella, Mucispirillum, and Parasutterella exhibited a negative correlation with phospholipids, whereas Enterobacter, Parabacteroides, Helicobacter, and Alistipes demonstrated a positive correlation with these compounds. Lachnospiraceae, Lachnoclostridium, and Alistipes positively interact with α-MCA and HDCA, while Faecalibaculum negatively interacts with these substances. These findings indicate a correlation between alterations in the gut microbiota and specific metabolites.

## Results of network proximity calculation

**Fig. 8:**
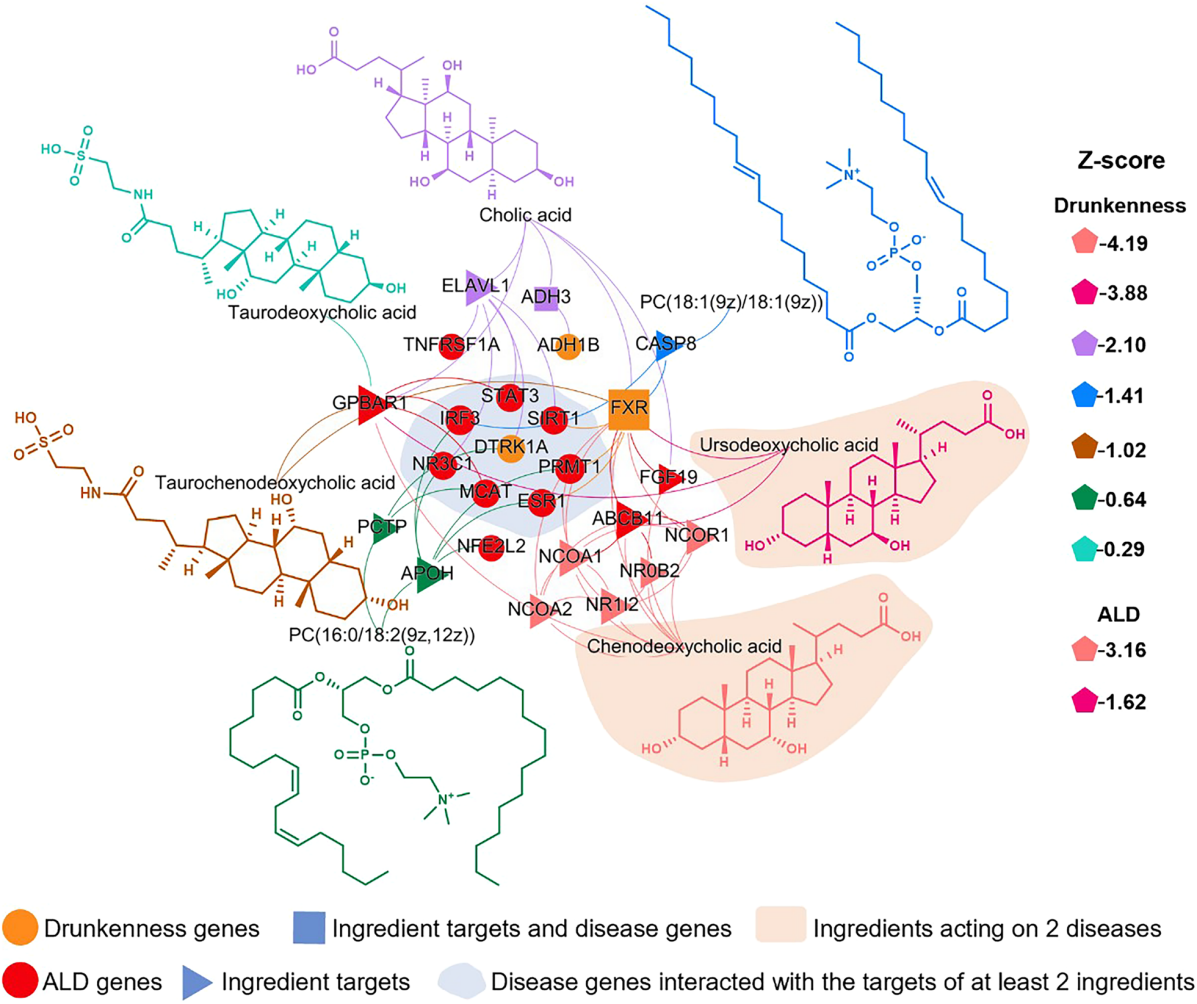
Network diagram of the potential active agents of GBP, targets, and disease-related genes. The size of each node reflects the number of connected component targets or disease-related genes. The color coding of the network is designated based on the categories of active ingredients and disease genes, whereas the size corresponds to the degree of connectivity (number of connections each node has). The shape of each node denotes its attribute within the network, with spatial proximity indicating similarity in interactions

Utilizing an integrative approach that combines database searches and extensive literature reviews, we curated a comprehensive list of agents, including 7 ingredients detected in serum and 39 metabolites, along with their corresponding targets (Additional file 3: Table S3). Additionally, we collected a list of 10 genes that cause drunkenness, enumerated in Additional file 4: Table S4, and 34 genes associated with ALD, presented in Additional file 5: Table S5. Subsequently, we mapped the agent targets and disease-related genes in the human protein–protein interaction network and calculated the network proximity between them (Additional files 6, 7: Table S6, S7). *Z*-closest < 0 and *P* < 0.05 were considered to indicate statistical significance. As shown in Fig. 8, the network encompasses 7 active agents, 3 genes associated with drunkenness, and 9 genes related to ALD. In total, the diagram features 32 nodes and 60 interactions. The color coding corresponds to the categories of bioactive agents and disease-associated genes, while the node size is indicative of the degree of connectivity. The nodal shape reflects the intrinsic properties of the nodes. Interestingly, the network medicine framework analysis indicated that chenodeoxycholic acid and ursodeoxycholic acid emerge as bioactive agents potentially modulating both drunkenness and ALD. Taurodeoxycholic acid, PC(16:0/18:2(9Z,12Z)), taurochenodeoxycholic acid, PC(18:1(9Z)/18:1(9Z)) and cholic acid were identified as potential active agents for reducing drunkenness. Among the genes potentially regulated by these bioactive agents, SIRT1, NR3C1, ESR1, IRF3, MCAT, PRMT1, and STAT3 are implicated, with DYRK1A being specifically associated with the genetic profile of drunkenness.

## Discussion

**Fig. 9:**
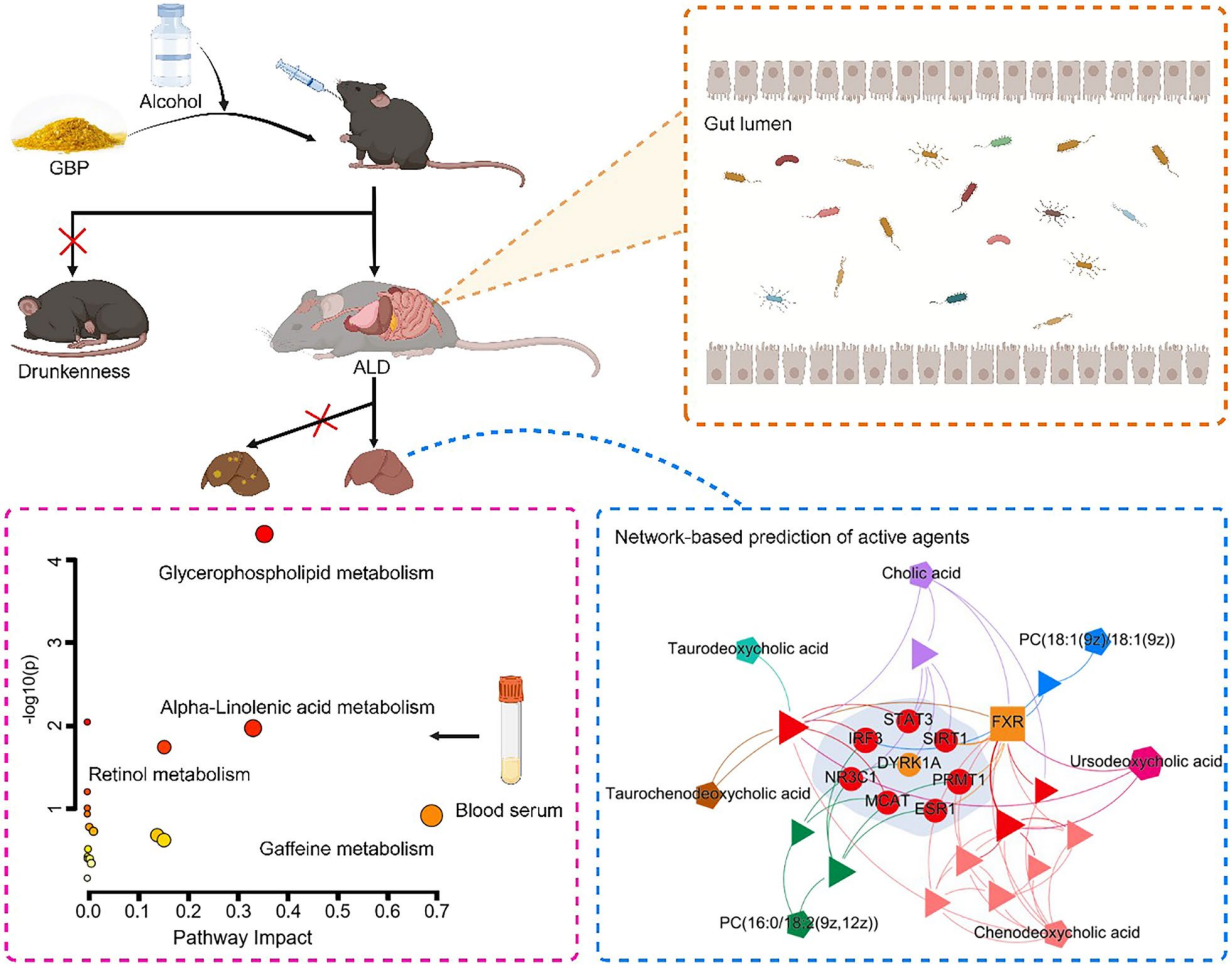
Illustration of the modulatory effects of GBP on the attenuation of drunkenness and ALD in mice. A schematic outlines the administration of GBP in mice and its subsequent inhibition of drunkenness and ALD symptoms (top left). A visual summary of the diversity of the gut microbiota within the intestinal lumen following GBP treatment (top right). A chart highlights the metabolic pathways altered after GBP treatment, notably glycerophospholipid metabolism, as suggested by the significant red marker (bottom left). The network diagram visualizes the complex interactions between bioactive agents, their molecular targets, and drunkenness or ALD genes. Nodes represent active agents that could play a role in modulating the impacts of alcohol (bottom right)

In this study, we report the interesting pharmacological benefits of GBP in ameliorating both behavioral and physiological symptoms associated with alcohol consumption, as summarized in Fig. 9. GBP administration significantly postponed the onset of drunkenness and reduced its duration in a mouse model. Furthermore, our study provides novel insights into the beneficial potential of GBP in modulating the gut-liver axis, as demonstrated by significant shifts in the gut microbiota composition and improved liver biochemical marker levels. Moreover, our findings further indicate the complex interplay between metabolomics profiles and liver function, supporting the notion that the beneficial impact of GBP extends to the restoration of microbial and metabolic homeostasis disrupted by alcohol. Network proximity calculations highlighted 7 and 2 potential active agents of GBP, reinforcing its therapeutic potential against both drunkenness and ALD, respectively. These novel discoveries contribute to the foundational evidence for the anti-drunkenness and hepatoprotective properties of GBP and may guide the development of innovative interventions for alcohol-induced conditions.

Previous research has shown that alcohol exposure in the intestines can perturb the gut microbiota [26]. In our current study, we found that the intestinal microbiome of vehicle-treated mice was characterized by a reduction in the abundance of phylum Lachnospiraceae and an enrichment of Escherichia-Shigella. Interestingly, GBP treatment significantly decreased the Escherichia-Shigella population, potentially mitigating the accumulation of pro-inflammatory substances such as lipopolysaccharides (LPS) [27, 28]. Simultaneously, we found that GBP treatment effectively increased the abundance of Lachnospiraceae, a group pivotal for producing short-chain fatty acids (SCFAs) essential for gut health [29]. These findings suggested that the gastrointestinal protective effects of GBP may stem from its ability to modulate the microbiota. Further correlation analyses revealed significant associations between gut bacteria and lipid metabolism. Specifically, the abundance of Escherichia-Shigella was positively correlated with the lipid indicators TC, TG, AKP, and ALT. In contrast, Lachnospiraceae exhibited relationships with TG, AKP, and ALT. In line with the findings of previous studies, gut bacteria substantially influence lipid metabolism and the inflammatory response, impacting intestinal barrier integrity and systemic lipid levels [30, 31].

Consistent with the literature, our results reinforce the notion that bile acid supplementation can modulate the expression of genes central to lipid metabolism, a process likely mediated through the activation of the nuclear bile acid receptor, FXR [5]. FXR activation is associated with improved insulin sensitivity, reduced triglycerides, lowered cholesterol, and suppressed hepatic inflammation [32]. Our network analysis extends these findings by identifying FXR as a target for a series of bile acids, including chenodeoxycholic acid, cholic acid, taurochenodeoxycholic acid, and ursodeoxycholic acid. In addition, there is a causal relationship between FXR and the pathology of ALD [33]. Hence, it is logical to postulate that GBP, which is composed principally of bile acids, may modulate lipid metabolism through FXR activation and indirectly by modifying the gut microbiota, which in turn might reduce alcohol-induced hepatic impairment.

Our analysis revealed that alcohol consumption leads to distinct alterations in serum metabolic profiles, with increased phospholipid and fatty acid levels in the alcohol-exposed group, echoing prior studies on the role of alcohol in upregulating fatty acid synthesis and consequent liver injury [34–36]. Intriguingly, supplementation with GBP was found to significantly counter these metabolic disruptions. In addition to fatty acid synthesis, alcohol consumption exerts a considerable effect on bile acid metabolism, which is typically characterized by an increase in biosynthetic-related gene expression and a concomitant decrease in hepatic uptake genes [37–39]. A reduction in taurine-conjugated bile acids, observed in the vehicle group, implied impaired hepatic clearance of free bile acids, a disruption that GBP treatment appeared to rectify.

We also noted a decrease in the levels of TCA and TDCA, alongside an increase in α-MCA, β-MCA, and HDCA in the vehicle group. This pattern suggested alcohol-induced expansion of the bile acid pool, a finding in agreement with previous reports [40]. The diminished presence of taurine-conjugated bile acids such as TCA and TDCA in systemic circulation could reflect the diminished efficacy of clearing free bile acids in the liver [41, 42]. GBP treatment was effective at alleviating these alcohol-induced metabolic perturbations. Furthermore, we found a notable negative correlation between the concentrations of α-MCA and HDCA and the activity of ALDH, an enzyme pivotal in metabolizing acetaldehyde to acetic acid during alcohol digestion [43].

The accumulation of acetaldehyde is one of the main causes of hangovers [44]. Notably, increased levels of α-MCA, β-MCA, and HDCA imply the potential exacerbation of hangovers and liver damage due to aberrant bile acid accumulation. Our correlation analysis revealed a multifaceted interaction between ALDH and the gut microbiota: ALDH was negatively correlated with Parasutterella, Mucispirillum, and Rikenella but was positively correlated with Helicobacter and Alistipes. Furthermore, Lactobacillus and Bifidobacterium strains in the gut can metabolize alcohol and acetaldehyde [45]. Furthermore, the activity of the gut microbiome profoundly impacts bile acid composition [46]. β-MCA is converted into ω-MCA through C-7 epimerization and then into HDCA via 7α-dehydroxylation, processes mediated by specific gut bacterial taxa [47–49]. Our findings align with existing research, indicating a positive correlation between the abundance of these bacterial genera and the serum levels of α-MCA and HDCA. In the GBP-treated group, gut microbiota restoration was accompanied by a decrease in bile acid levels, suggesting the influence of GBP on the intestinal flora composition and, consequently, bile acid metabolism [28]. However, the interplay between bile acids and the intestinal microbiota remains intricate, warranting further exploration to fully decipher these relationships.

Our study sheds light on the capacity of network medicine to decode the intricate interplay in biological systems, emphasizing the necessity for an integrated approach in drug development and therapeutic assessment for ALD. This shift toward a systems biology perspective in understanding disease mechanisms and evaluating treatment efficacy provides hope for innovative therapeutic approaches. However, it is crucial to acknowledge the limitations of our research. While network medicine methodologies have helped predict the active constituents of GBP, the exact bioactive molecules and their mechanistic pathways require further experimental validation. Future research should focus on isolating and confirming the predicted active compounds in GBP, examining their principal effects, investigating the potential synergistic outcomes of combining these ingredients, and understanding their mechanisms of action.

## Conclusions

This study revealed that dietary supplementation with GBP prevents both drunkenness and ALD in mice. This protective effect appears to be mediated by the modulation of the gut microbiota and the amelioration of metabolic disturbances. Employing a network medicine approach, our analysis identified five key agents, namely taurodeoxycholic acid, PC(16:0/18:2(9Z,12Z)), taurochenodeoxycholic acid, PC(18:1(9Z)/18:1(9Z)), and cholic acid, as pivotal for alleviating drunkenness. Furthermore, two agents, chenodeoxycholic acid and ursodeoxycholic acid, were identified as key agents for combating both drunkenness and ALD. The integration of microbiota analysis, metabolomics and network medicine highlights the innovative potential of traditional Chinese medicine in developing new therapeutic strategies for managing drunkenness and ALD. This could set the stage for future breakthroughs in treatment approaches.

### Supplementary Information

**Additional file 1: Table S1.** A list of identified ingredients in mouse serum after GBP treatment.

**Additional file 2: Table S2.** A list of up-regulated metabolites in mouse serum after GBP treatment.

**Additional file 3: Table S3.** List of agents and targets.

**Additional file 4: Table S4.** Drunkenness-associated genes.

**Additional file 5: Table S5.** ALD-associated genes.

**Additional file 6: Table S6.** Network proximity scores of potential active agent-drunkenness associations.

**Additional file 7: Table S7.** Network proximity scores of potential active agent-ALD associations.

## Data Availability

The datasets and all the relevant codes are available from the corresponding author.

## Abbreviations

ALD: Alcoholic liver disease
ALT: Alanine aminotransferase
ALDH: Acetaldehyde dehydrogenase
AST: Aspartate aminotransferase
AKP: Alkaline phosphatase
GBP: Golden bile powder
SOD: Superoxide dismutase
TC: Total cholesterol
TG: Triglycerides
